# Androgen receptor variant 7 exacerbates hepatocarcinogenesis in a c-MYC-driven mouse HCC model

**DOI:** 10.1038/s41389-023-00449-3

**Published:** 2023-02-06

**Authors:** Tatsuo Kido, Yun-Fai Chris Lau

**Affiliations:** 1 Division of Cell and Developmental Genetics, Department of Medicine, San Francisco VA Health Care System, University of California, San Francisco, CA USA; 2 Institute for Human Genetics, University of California, San Francisco, CA USA; 3 Liver Center, University of California, San Francisco, CA USA

**Keywords:** Oncogenes, Cancer genomics, Cancer genetics

## Abstract

Androgen receptor variant 7 (AR-V7), an AR isoform with a truncated ligand-binding domain, functions as a transcription factor in an androgen-independent manner. AR-V7 is expressed in a subpopulation of hepatocellular carcinoma (HCC), however, its role(s) in this cancer is undefined. In this study, we investigated the potential roles of AR-V7 in hepatocarcinogenesis in vivo in a c-MYC-driven mouse HCC model generated by the hydrodynamic tail-vein injection system. The impacts of AR-V7 on gene expression in mouse HCC were elucidated by RNA-seq transcriptome and ontology analyses. The results showed that AR-V7 significantly exacerbated the c-MYC-mediated oncogenesis in the livers of both sexes. The transcriptome and bioinformatics analyses revealed that AR-V7 and c-MYC synergistically altered the gene sets involved in various cancer-related biological processes, particularly in lipid and steroid/sterol metabolisms. Importantly, AR-V7 suppressed a tumor suppressor Claudin 7 expression, upregulated by c-MYC overexpression via the p53 signaling pathway. Claudin 7 overexpression significantly suppressed the c-MYC-driven HCC development under p53-deficient conditions. Our results suggest that the AR-V7 exacerbates the c-MYC-driven hepatocarcinogenesis by potentiating the oncogenic roles and minimizing the anti-oncogenic functions of c-MYC. Since AR-V7 is expressed in a subpopulation of HCC cases, it could contribute to the inter- and intra-heterogeneity of HCC.

## Introduction

Primary liver cancer is the third leading cause of cancer death worldwide with more than 900,000 new cases and 800,000 deaths every year [1]. Primary liver cancer comprises hepatocellular carcinoma (HCC) (~80% of cases), intrahepatic cholangiocarcinoma (iCC) (~15% of cases), and other rare subtypes including hepatoblastoma [1]. Various oncogenic pathways, including the c-MYC pathway, are complexly involved in HCC development, leading to high inter- and intra-tumor heterogeneity [2, 3]. Such complexity has complicated the development of treatment strategies for HCC. c-MYC is a master regulator of various biological processes, such as cell proliferation, differentiation, metabolism, and apoptosis, serving both normal physiology and, when aberrantly activated, could promote oncogenesis in many organs/tissues in a context-dependent manner(s). It functions as a proto-oncogenic transcription factor contributing to the initiation and progression of a wide variety of cancer types [4–6]. In HCC, the c-MYC gene is amplified in ~30% cases [4] and the c-MYC signaling could be potentiated in the absence of any alteration of the c-MYC gene [7]. Further, HBx, an oncoprotein encoded by the hepatitis B virus (HBV) genome, activates the c-MYC gene in the liver of patients with hepatitis B infection [8]. Similarly, hepatitis C virus (HCV) infection upregulates the c-MYC expression in HCC cell lines [9]. Since >75% of HCC cases were attributed to HBV and/or HCV infections [1], the c-MYC pathway could contribute widely to HCC development. However, some studies using mouse models showed that solo hepatocytic overexpression of c-MYC in adult mice was not sufficient to initiate or promote cancer after a prolonged latency [3, 10, 11]. Indeed, as the c-MYC pathway is often activated along with other oncogenic pathways in clinical HCC, recent studies have identified various pathways, such as AKT, YAP/TAZ, β-catenin, and RAS pathways, which could collaborate with the c-MYC pathway and exacerbate the c-MYC-mediated oncogenic processes [3, 12–14]. Thus, the identification of novel factors synergistically exacerbating the c-MYC pathway in hepatocarcinogenesis could provide significant insights into the mechanisms of tumor heterogeneity, thereby improving treatment options in liver cancer.

**Fig. 1:**
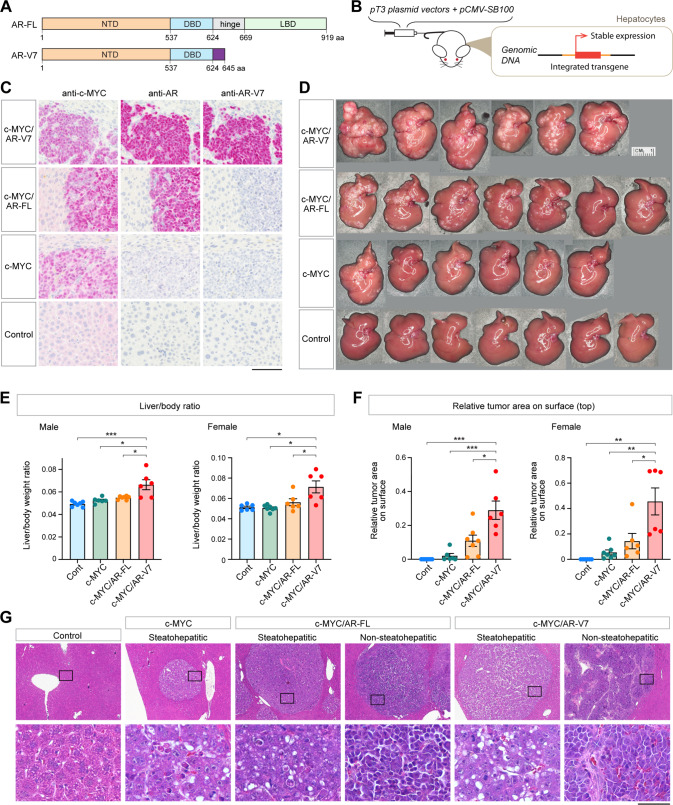
AR-V7 and AR-FL differentially exacerbated the c-MYC-driven mouse HCC development. **A** Schematic representation of the human androgen receptor structures of full-length AR (AR-FL) and AR-V7 variant. NTD N-terminal transactivation domain, DBD DNA binding domain, LBD ligand-binding domain. **B** Schematic illustration of the hydrodynamic injection for hepatocytic stable transfection. DNAs inserted in pT3 vector are integrated into the genome via the Sleeping Beauty transposon system and stably expressed in the transfected hepatocytes. **C** Verification of the expression of c-MYC, AR-FL, and AR-V7 in the mouse liver specimens by immunohistochemistry (red) at 20 dpi of the expression vectors of c-MYC and AR-V7 (c-MYC/AR-V7), c-MYC and AR-FL (c-MYC/AR-FL), c-MYC alone (c-MYC), or an empty vector (Control). Nuclei were visualized by hematoxylin staining (blue). Scale bar = 100 μm. See Materials and Methods for specific antibodies used. Anti-AR monoclonal antibody targets the N-terminal domain, present in both AR-FL and AR-V7. **D** Macroscopic phenotypes of the liver in male mice of indicated groups at 20 dpi. **E**, **F** Liver to body weight ratio (**E**) and relative surface tumor areas (**F**) in male and female mice at 20 dpi. Liver samples presented in **D** (male) and Supplementary Fig. 3 (female) were analyzed as described in the Materials and Methods. Control, *n* = 7 (male) or 6 (female); c-MYC, *n* = 6 (male) or 7 (female); c-MYC/AR-FL, *n* = 7 (male) or 6 (female); c-MYC/AR-V7, *n* = 6 (male and female). Statistical significance by one-way ANOVA with Tukey’s multiple comparisons test; *p < 0.05; **p < 0.001; ***p < 0.0001. Error bars indicate mean ± SEM. **G** Microscopic images of H&E-stained tissue section of male mouse liver of indicated groups at 20 dpi. Bottom panels show magnified images of the boxed areas in the top panels. Scale bar = 400 or 50 μm.

Androgen receptor (AR) is the male sex hormone receptor and a transcription factor playing pivotal roles in the development of male reproductive organs and masculinization of various somatic organs, including the central nervous system [15, 16]. The full-length AR (AR-FL) consists of an N-terminal domain (NTD), DNA binding domain (DBD), and C-terminal ligand-binding domain (LBD), and forms transcriptional complexes with co-factors and binds on its target genes to regulate their expression in a ligand-dependent manner (Fig. 1A) [16, 17]. AR also exerts pathogenic functions in various cancer types, particularly in prostate cancer [16, 17]. Thus, androgen deprivation therapy (ADT) is one of the first-line treatment strategies for localized and metastatic prostate cancer [16, 17]. However, AR splicing variants lacking the ligand-binding domain, particularly AR variant 7 (AR-V7) that harbors the small CE3 domain instead of the LBD (Fig. 1A and Supplementary Fig. 1A), functions in a ligand-independent manner and could contribute to the development/progression of castration-resistant prostate cancer with poor clinical outcomes [18–20]. Since AR-V7 functions in an androgen-independent manner, it could contribute to cancer development in men and women regardless of androgen levels in other cancer types. Indeed, recent studies revealed that AR-V7 is expressed in breast cancer and associated with cancer metastasis [21, 22]. Noticeably, analyses of public databases, such as TCGA, showed that AR-V7 is potentially expressed in some HCC cases at comparable levels to those in prostate cancer (Supplementary Fig. 1B and Supplementary Table 1). Consistently, our preliminary study by conventional RT-PCR analyses showed that 5 out of 13 HCC samples (38%) expressed AR-V7 (Supplementary Fig. 1C). Hence, a subpopulation of HCC cases might also be affected by the heterogeneously expressed AR-V7. One previous study demonstrated the expression of AR variants, including AR-V7, in HCC patients in TCGA datasets, pathological HCC samples, and some established HCC cell lines, and showed that overexpression of AR-V7 could contribute to invasion of HCC cell lines, rather than cell proliferation [23]. However, the roles of AR-V7 in hepatocarcinogenesis under in vivo conditions need to be investigated.

Since the c-MYC pathway is frequently and aberrantly activated in HCC, we investigated the roles of AR-V7 in hepatocarcinogenesis in vivo by utilizing a c-MYC-driven mouse HCC model. The roles of AR-FL were also investigated simultaneously. The c-MYC-driven HCC model mice that co-expressed AR-V7 or AR-FL were generated using the hydrodynamic tail-vein injection technique and the Sleeping Beauty transposon system [13, 24]. The results showed that co-expression of AR-V7 with c-MYC significantly exacerbated HCC development in both male and female mice, compared with those with c-MYC overexpression alone. Co-expression of AR-FL resulted in moderate exacerbation in male mice, but not in female mice. Further, RNA-seq transcriptome analyses showed that AR-V7 potentiated several cancerous c-MYC downstream genes and suppressed a tumor suppressor Claudin 7 (Cldn7) that was upregulated by c-MYC overexpression. Importantly, preliminary expression analyses confirmed that c-MYC and AR-V7 were co-expressed in a subpopulation of clinical HCC cases (Supplementary Fig. 1C). In addition, data mining of TCGA datasets showed a weak but significant positive correlation between c-MYC and AR exon-CE3 expressions in HCC cases (Supplementary Fig. 1D), suggesting a potential interaction between these genes. Our study demonstrated a potential mechanism(s) on how AR-V7 contributes to hepatocarcinogenesis and cancer heterogeneity in HCC.

## Materials and methods

### Mouse model of liver cancer using hydrodynamic tail-vein injection

The pT3-based c-MYC expression vector pT3-c-MYC (#92046) [25], the SB100 transposase expression vector pCMV-SB100 (#34879) [26], the CRISPR/Cas9-base mouse p53 knockout vector pX330-p53 (#59910) [27], and the full-length human androgen receptor vector pLENTI6.3/AR-GC-E2325 (#85128) were obtained from the Addgene Repository (Watertown, MA). The pT3-EF1α vector and the AR-V7 cDNA were kindly provided by Dr. Xin Chen (University of Hawaii) and Dr. Yun Qiu (University of Maryland School of Medicine) [20], respectively. The mouse Cldn7 cDNA was generated by RT-PCR using the Cldn7 primers 5′-GCAAGCTTGACAAGGAAATGGCCAACTCG-3′ and 5′-GCGTCGACTCACACGTATTCCTTGGAGGA-3′, cloned into a cloning vector pMiniT2.0 (New England Biolabs, Ipswich, MA), and confirmed by DNA sequencing. The pT3-AR-V7, pT3-AR-FL, and pT3-Cldn7^FLAG^ vectors were generated in-house, using pDONR221, pENTR-1A, pT3-EF1α (used as a backbone), and the Gateway cloning system (Thermo Fisher Scientific, Waltham, MA) (Supplementary Fig. 2).

The 5- to 6-weeks-old FVB/N mice (Jackson Laboratory, Bar Harbor, ME) were divided randomly into groups of 6–7 animals for each sex, and hydrodynamic tail-vein injection was performed as described previously [24, 25]. The sample size was determined to detect at least 15% changes in tumor size/area with 90% power and a significance level of 5% [28]. In brief, 10 μg pT3-c-MYC, 10 μg pT3-AR-FL, 10 μg pT3-AR-V7, and/or 10 μg pT3-Cldn7^FLAG^ were diluted with 2 μg pCMV-SB100 in 2 mL saline (0.85% NaCl), sterilized through 0.2-μm filter and injected into the lateral tail-vein of a recipient mouse (20 g body size) in 7 seconds. Total amount of injected DNA was adjusted using pT3-EF1α as a control null vector. Animals were monitored every 2 days for tumor growth, harvested at 20 days post-injection for analyses. The Institutional Animal Care and Use Committee approved all experimental procedures accordingly to the NIH Guide for Care and Use of Laboratory Animals.

### Immunohistochemistry and immunofluorescence

Immunohistochemistry and immunofluorescence were performed as described previously [29], using an anti-c-MYC rabbit monoclonal antibody (clone Y69, Abcam #ab32072, Cambridge, MA), anti-AR[N-terminal] (AR-NTD) rabbit monoclonal antibody (clone ER179(2), Abcam #ab108341), anti-AR-V7 rabbit monoclonal antibody (clone RM7, ReMAb Biosciences #31-1109-00, South San Francisco, CA), and anti-FLAG tag mouse monoclonal antibody (clone M2, Sigma-Aldrich #F1804). The immunoreactive signals were visualized either by the VECTASTAIN ABC-AP kit (Vector Laboratories, Burlingame, CA) or by Alexa Fluor 594 (red) conjugated anti-rabbit immunoglobulin G (Molecular Probes # A21207, Thermo Fisher Scientific, Waltham, MA) and Alexa Fluor 488 (green)-conjugated anti-mouse immunoglobulin G (Molecular Probes #A11029). Nuclear DNA was visualized by staining with 4’,6-Diamidine-2’-phenylindole dihydrochloride (DAPI) (Roche Applied Science).

### Image data analysis

Surface areas of the liver and liver cancer in photographic images were manually selected and quantified using the ImageJ program (version 1.52a) (https://imagej.nih.gov/ij/) as shown in Supplementary Fig. 3. The relative cancer area referred to the whole liver surface was calculated and statistically analyzed using the Prism 9 program (GraphPad Software, San Diego, CA).

### RNA-seq transcriptome analysis

At 20 days post hydrodynamic injection, liver tumor foci were dissected and pooled for each mouse. Non-tumor liver tissues were collected from the control group mice. Total RNA was isolated from tumor foci and non-tumor tissues using TRIZOL-Plus RNA isolation kit (Thermo Fisher Scientific, Waltham, MA). One μg total RNA from each biological triplicate or quadruplicate sample was subjected to a total mRNA sequencing (RNA-seq) and subsequent data analyses with the Ensembl GRCm39 (release M27) mouse reference genome, as described previously [30]. Genes with a failure discovery ratio (FDR) < 0.05, Student’s *t* test *P* value <0.05, and fold change >2, compared to the sex-matched control group were selected as significant differentially expressed genes (DEGs) in bioinformatic analyses. Gene ontology and pathway analyses were performed using the Database for Annotation, Visualization, and Integrated Discovery (DAVID) bioinformatics resources (version 2022) [31].

### Quantitative RT-PCR

Total RNA isolated from mouse tissue samples was treated by RQ1-DNase treatment (Promega, Madison, WI) to remove contaminant DNA and was reverse-transcribed using the SuperScript IV reverse-transcriptase kit (Thermo Fisher Scientific). The reverse-transcribed products were analyzed by quantitative PCR (RT-qPCR) in biological triplicate or quadruplicate for each sample using TaqMan Fast Advanced Master Mix (Thermo Fisher Scientific) and QuantStudio3 real-time PCR detection system (Thermo Fisher Scientific). The TaqMan gene expression assay IDs are described in Supplementary Table 2. The Gapdh gene was used as an internal control for normalization. The relative change referred to the sex-matched control group was calculated and statistically analyzed using the Prism 9 program.

### Conventional RT-PCR and sequencing analysis

De-identified human frozen HCC and cirrhotic liver samples were obtained from the Cooperative Human Tissue Network (CHTN). Consents from donors were obtained by CHTN at the time of procurement, per the established policy of CHTN. RNA isolation and conventional RT-PCR analysis were performed using the primers listed in Supplementary Table 3 as previously described [29]. The studies were performed under an exempted protocol, approved by the Institutional Committee on Human Research. The RT-PCR products were analyzed by electrophoresis, cloned into the pGEM-T Easy vector (Promega), sequenced with an automatic sequencer at Elim Biopharmaceuticals (Hayward, CA), and analyzed by the MacVector Program (MacVector, Apex, NC).

## Results

### AR-V7 exacerbated the c-MYC-driven HCC development similarly in both male and female mice

Since the c-MYC pathway has been demonstrated to significantly contribute to HCC oncogenesis, we evaluated the effects of AR-V7 expression on the c-MYC-driven hepatocarcinogenesis in vivo. To generate the mouse model, we stably transfected the c-MYC expression vector pT3-c-MYC with the AR-V7 expression vector pT3-AR-V7 (hereby designated as hep-c-MYC/AR-V7) or without pT3-AR-V7 (designated as hep-c-MYC) into mouse hepatocytes using the hydrodynamic tail-vein injection technique (Fig. 1B). We also generated the hep-c-MYC/AR-FL mice and control mice similarly by hydrodynamic injection of the pT3-c-MYC and pT3-AR-FL or pT3-EF1α empty vector, respectively. The expressions of c-MYC, AR-V7, and AR-FL in the developed tumor foci were confirmed by immunohistochemistry at 20 days post-injection (dpi) (Fig. 1C). When AR-V7 alone was injected in the adult mice, no tumor was observed in their livers up to 20 dpi. However, AR-V7 expression was observed in selected hepatocytes at 3 dpi but was barely detectable at 20 dpi (Supplementary Fig. 4).

At 20 dpi, the overall tumor volume in the hep-c-MYC/AR-V7 mice was larger than hep-c-MYC mice regardless of the sexes as indicated by increased liver-to-body weight ratio (Fig. 1D, E; Supplementary Fig. 3). Similarly, the relative tumor area was larger in hep-c-MYC/AR-V7 mice than hep-c-MYC mice in both sexes (Fig. 1F and Supplementary Fig. 3). These results suggest that AR-V7 potentiated the c-MYC-driven hepatocarcinogenesis in a ligand-independent manner.

Histopathologic analyses of hematoxylin-eosin (H&E) stained sections revealed that tumors developed in hep-c-MYC mice were moderately steatohepatitic, contained small droplets of fat in cancer cells (Fig. 1G, c-MYC). On the other hand, tumors developed in hep-c-MYC/AR-V7 mice were morphologically heterogenous, with steatohepatitic and non-steatohepatitic foci regardless of the sexes (Fig. 1G, c-MYC/AR-V7). Cells in non-steatohepatitic tumor foci had scant cytoplasm without droplets of fat (Fig. 1G, c-MYC/AR-V7).

On the other hand, the relative tumor area in hep-c-MYC/AR-FL male mice were morphologically larger than that in hep-c-MYC male mice (Fig. 1F and Supplementary Fig. 3). In addition, histopathologic analyses showed that non-steatohepatitic tumor foci were developed in male c-MYC/AR-FL mice similar to hep-c-MYC/AR-V7 mice (Fig. 1G, c-MYC/AR-FL). In contrast, non-steatohepatitic tumor foci were not developed in female c-MYC/AR-FL mice and relative surface tumor area was not obviously different from that of female hep-c-MYC mice (Fig. 1F and Supplementary Fig. 3). These results indicate that AR-FL could also exert mild effects, albeit at much lower levels than those for AR-V7, on c-MYC-driven hepatocarcinogenesis in males but not females, suggesting a male-biased exacerbation of the resulting HCC between the sexes.

### Transcriptome analyses revealed the biological processes that were synergistically altered by c-MYC and AR-V7

**Fig. 2:**
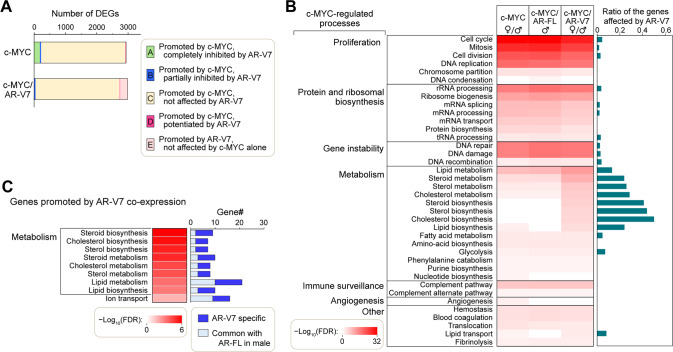
Effect of the co-expression of AR-V7/AR-FL on the gene expression profiles of the c-MYC-driven liver cancer. **A** Number and classification of the differentially expressed genes (DEGs) in mouse HCC promoted by c-MYC alone (c-MYC) or c-MYC and AR-V7 co-expression (c-MYC/AR-V7) at 20 dpi (class A-E). AR-V7 affected only subsets of c-MYC-mediated DEGs. **B** Results of DAVID functional enrichment analyses for the DEGs in the mouse HCC samples of hep-c-MYC (common between male and female), hep-c-MYC/AR-FL (male), or hep-c-MYC/AR-V7 (common between male and female). Selected categories of the c-MYC-regulated processes are indicated on left. Right panel indicates the portions of genes in each biological process affected by AR-V7, with the most effects being in those in metabolism. **C** Metabolic processes promoted by AR-V7 (dark blue) and shared with AF-FL (light blue) co-expression with c-MYC, respectively.

The c-MYC plays significant roles in many biological processes, including proliferation, protein and ribosomal biosynthesis, metabolism, immune surveillance, cell differentiation, cell adhesion, and senescence. When it is aberrantly overexpressed/activated, it amplifies the biological processes toward oncogenesis, such as gene instability, accelerated cell proliferation, metabolism, and angiogenesis, resulting in cancer development [4]. To explore the likely mechanisms by which AR-V7 potentiates the c-MYC-driven hepatocarcinogenesis, we performed a gene expression profiling of liver cancer developed in hep-c-MYC and hep-c-MYC/AR-V7 mice at 20 dpi by RNA-seq transcriptome analyses. Liver samples from mice at 20 dpi injected with an empty vector alone were used as controls. Since AR-V7 and c-MYC cooperatively promoted liver cancer regardless of the sexes, we identified the genes that were commonly altered in male and female mice. The results showed that the expression patterns of 2930 genes were differentially altered by c-MYC overexpression (Supplementary Table 4). Out of these, 2691 genes were not further affected by AR-V7 co-expression (class-C, yellow in Fig. 2A). AR-V7 co-expression potentiated the c-MYC-mediated changes of 17 genes (class-D, red in Fig. 2A), completely (class A) and partially (class-B) repressed 181 and 41 c-MYC-regulated genes respectively (green and blue in Fig. 2A). In addition, expression patterns of 243 genes were affected by AR-V7 co-expression but not by c-MYC alone (class-E, light red in Fig. 2A), and hence could be attributed to AR-V7 effects under such conditions. The functional annotation enrichment analysis using the DAVID bioinformatics resources [31] revealed that various c-MYC-regulated biological processes, including proliferation, protein and ribosomal biosynthesis, and gene instability were significantly affected, while metabolism and other pathways were further altered by AR-V7 co-expression at various levels (Fig. 2B). Processes in metabolism were mostly affected by the AR-V7 co-expression (Fig. 2B, right panel). An enrichment analysis that focused on the genes potentiated or promoted by AR-V7 co-expression (dark-red and light-red areas in Fig. 2A) suggested that major portions of the genes involved in lipid metabolism and steroid/sterol metabolism were significantly affected by AR-V7 (Fig. 2C).

The macroscopic analyses showed that AR-FL co-expression moderately exacerbated the c-MYC-driven liver cancer in male mice, but not in female mice (Fig. 1F). Hence, we further investigated the gene expression profiles and enriched biological processes in the male hep-c-MYC/AR-FL liver cancer. The results showed that the AR-FL could share portions of the gene sets in metabolism processes affected by the AR-V7 co-expression (Fig. 2C, right panel), suggesting that AR-FL exacerbates, at a reduced level, certain c-MYC-driven hepatocarcinogenic processes in male mice similar to those of AR-V7.

**Fig. 3:**
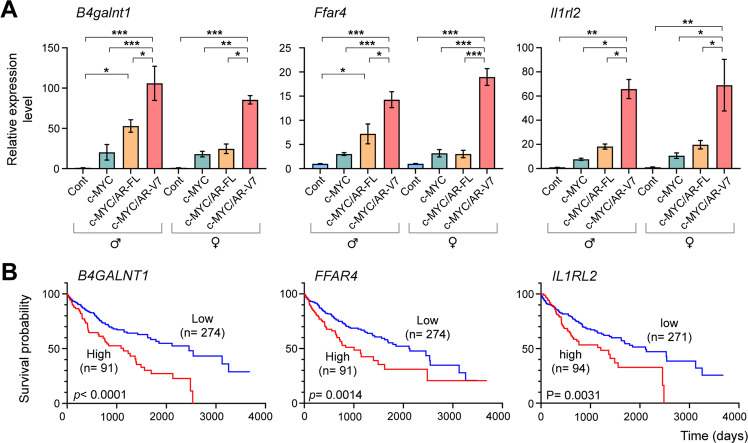
Examples of three genes upregulated by AR-V7 co-expression in the c-MYC-driven mouse HCC models and the corresponding survival patterns in HCC patients. **A** Quantitative RT-PCR for expressions of B4galnt1, Ffar4, or Il1rl2, in respective sample groups. The *Y* axis indicates the expression levels relative to Gapdh. Statistical significance by one-way ANOVA with Tukey’s multiple comparisons test; *p < 0.05; **p < 0.001; ***p < 0.0001. Error bars indicate mean ± SEM. **B** Kaplan–Meier survival plots of HCC patients in TCGA datasets for the indicated genes. Red line indicates high expressor and blue line indicates low expressor, respectively. Log-rank test *P* value is indicated.

To evaluate the effects of AR-V7 on c-MYC-driven hepatic oncogenesis, we examined specific genes, i.e., B4galnt1/G4GALNT1, Ffar4/FFAR4, and Il1rl2/IL1RL2, that was further upregulated by AR-V7 co-expression, as indicated in class-D genes (Fig. 2A, red). The corresponding survival patterns in HCC patients with high and low expression of the respective class-D genes in the TCGA database were analyzed. These genes were reported in the literature to be associated with oncogenesis of various human cancers. The B4galnt1 gene encodes the enzyme beta-1,4-N-acetylgalactosaminyl transferase, involved in the biosynthesis of complex gangliosides. Various studies demonstrated that B4GALNT1 was involved in the progression of different cancer types including oral squamous cell carcinoma and lung adenocarcinoma [32, 33]. Quantitative RT-PCR analysis showed that it was upregulated by c-MYC but was further elevated by AR-V7 co-expression in both male and female HCC samples (Fig. 3A, far left panel). Its expression level was negatively correlated with the survival ratio in HCC patients (Fig. 3B, far left panel). Several other genes, including the free fatty acid receptor 4 (Ffar4), and interleukin 1 receptor like 2 (Il1rl2), also showed similar expression patterns in our mouse model and their high levels of expression correlated with poor clinical outcomes in HCC patients (Fig. 3A, B). FFAR4 has been demonstrated to promote epithelial-mesenchymal transition, cell proliferation/migration, and drug resistance in various cancer types [34]. IL1RL2 (also known as Interleukin 36 receptor, IL36R) is involved in tissue fibrosis and metastatic potential in breast and colon cancers [35, 36]. Our results showed that AR-V7 could upregulate the expression of these pro-oncogenic genes, and directly or indirectly potentiate the c-MYC-related oncogenic processes, resulting in exacerbation of the c-MYC-driven HCC. AR-FL co-expression showed moderate increases in the expression of these pro-oncogenic genes (Fig. 3A, B) and could contribute similarly to the exacerbation of the c-MYC-driven liver cancer development in males, but at a much-reduced level. We surmise that such similarities could contribute to the relatively milder exacerbation of c-MYC-driven hepatocarcinogenic processes in hep-c-MYC/AR-FL male mice than those of hep-c-MYC/AR-V7 mice. On the other hand, genes affected at higher levels and/or unique to AR-V7 actions could be responsible for the high exacerbation effects in c-MYC-driven hepatic oncogenesis in the hep-c-MYC/AR-V7 mice.

### The tumor suppressor Claudin 7 is a potentially key target of AR-V7 in the hep-c-MYC/AR-V7 HCC

**Fig. 4:**
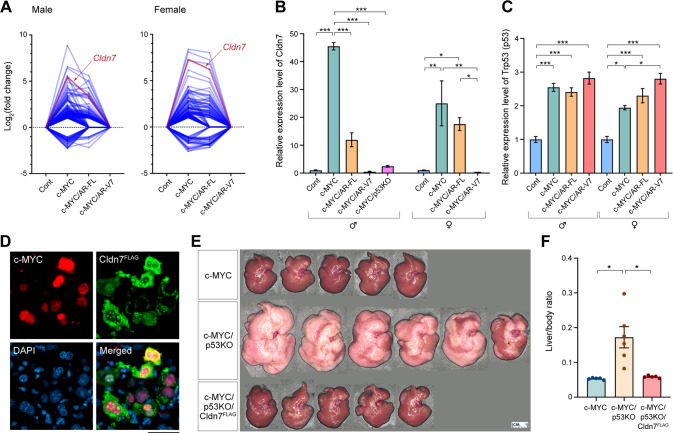
AR-FL and AR-V7 differently suppressed a tumor suppressor gene Cldn7 that was upregulated by c-MYC overexpression in mouse HCC models. **A** RNA-seq transcriptome analysis showed that 150 genes that were upregulated by c-MYC were repressed by AR-FL and/or AR-V7 co-expression; and 31 genes that were downregulated by c-MYC were upregulated by AR-FL and/or AR-V7 co-expression in both male (left) and female (right) mice at 20 dpi respectively. Each blue line represents differential expression levels (fold change in reference to control, *Y* axis) in the tumors of the respective combination of injected gene(s), *X* axis. Red lines highlight the differential expression levels of Cldn7 in respective samples. **B** Quantitative RT-PCR for expressions of Cldn7 in respective groups at 20 dpi. Expression of Cldn7 in male mice of c-MYC/p53KO (hep-c-MYC/p53KO) tumor at 11 dpi (pink bar), indicating that Cldn7 was repressed in p53-deficient conditions and hence was likely regulated by p53 [70–72]. **C** Expression of p53 in respective mouse groups in male and female, indicating p53 was upregulated by c-MYC but not significantly affected by AR-FL or AR-V7 co-expression. **D** Immunofluorescent images of c-MYC (red), Cldn7^FLAG^ (green), and DNA (blue) in the liver of hep-c-MYC/p53KO/Cldn7^FLAG^ mouse at 2 dpi, showing co-expression of the respective transgenes in the same cells. Scale bar = 50 µm **E**, Macroscopic phenotypes of the livers in male mice at 11 dpi injected with the expression vectors of c-MYC (c-MYC) (*n* = 5), c-MYC under p53-deficient condition (c-MYC/p53KO) (*n* = 6), or c-MYC and Cldn7^FLAG^ under p53-deficient condition (c-MYC/p53KO/Cldn7^FLAG^) (*n* = 5). **F** Liver to body weight ratio of the samples presented in **E**, indicating that the exacerbation of c-MYC-driven HCC under p53-deficient conditions was completely reversed by co-expression of Cldn7^FLAG^. Statistical significance by one-way ANOVA with Tukey’s multiple comparisons test for **B**, **C**, and **F**; *p < 0.05; **p < 0.001; ***p < 0.0001. Error bars indicate mean ± SEM.

While c-MYC plays pivotal roles in cell proliferation and cancer development, it also increases the sensitivity to apoptosis in non- and pre-malignant cells [37, 38]. Further, hepatocytic c-MYC overexpression promoted liver cancer depending on the developmental context, and adult mice showed a longer latency to develop cancer than newborn mice due to activation of the tumor suppressor p53 [11]. Hence, we speculated that AR-V7 might also reduce the c-MYC-mediated anti-proliferative pathways in the transfected hepatocytes, in addition to potentiating the c-MYC-mediated oncogenic pathways. By exploring the transcriptome data of the 222 c-MYC downstream genes whose expression levels were partly (41 genes) or completely (181 genes) repressed by AR-V7 co-expression (green and blue areas in Fig. 2A and Supplementary Table 4), we found that the tumor suppressor gene Claudin 7 (Cldn7) was upregulated more than 40 folds in hep-c-MYC cancer compared to control liver, whereas such upregulation was reversed in the hep-c-MYC/AR-V7 cancer in both male and female mice (Fig. 4A). Such differential expression was confirmed by quantitative RT-PCR analysis (Fig. 4B). Noteworthy, the c-MYC-induced Cldn7 upregulation was partly repressed by the AR-FL-co-expression in male mice, but not in female mice (Fig. 4B). These results suggest that c-MYC upregulation of the Cldn7 tumor suppressor gene is completely and partially repressed by AR-V7 and AR-FL in a ligand-independent and ligand-dependent manner respectively.

Claudin 7 is a member of the claudin family that is involved in wide variety of biological processes including tight junction formation, cell polarity, signal transduction, transcription regulation, and mRNA stability [39, 40]. Recent studies revealed that the human CLDN7 is upregulated by the p53 signaling pathway and suppresses cell proliferation in various cancer types [41, 42]. Hence, we investigated further the expression and function of Cldn7 in the c-MYC-driven HCC mouse model. First, to explore the correlation between p53 and Cldn7 genes in the hep-c-MYC cancer, we analyzed the effect of the p53-deficiency on the Cldn7 expression in the tumors promoted by the c-MYC overexpression. When c-MYC was overexpressed under the p53-deficient condition (hep-c-MYC/p53KO) by co-injection of pX330-p53, the CRISPR base p53 knockout vector [27], cancer developed rapidly, as compared to those promoted by c-MYC alone. The pX330-p53 is a single plasmid vector capable of inactivating/deleting the p53 gene in the cells via the CRISPR targeting mechanisms. The inactivated p53 gene produces non-functional transcripts (confirmed as the dominant transcripts by sequencing, Supplementary Fig. 5), distinguishable from the wild-type transcript. The tumor size reached the end-point criteria at 11 dpi. A quantitative RT-PCR analysis showed that the expression level of Cldn7 in hep-c-MYC/p53KO cancer (*n* = 4, male mice) at 11 dpi was significantly lower than that of hep-c-MYC cancer at 20 dpi (Fig. 4B, c-MYC/p53KO). These results suggest that the Cldn7 gene is upregulated by c-MYC in a p53-dependent manner, thereby confirming that Cldn7 is a target of p53 regulation [41, 42]. Although p53 was upregulated in the hep-c-MYC liver cancer samples (Fig. 4C), the AR-V7 co-expression did not significantly affect the p53 expression level (Fig. 4C), suggesting that AR-V7 might directly suppress the Cldn7 expression downstream of p53.

Next, we evaluated the impact of Cldn7 expression on the c-MYC-driven HCC development under p53-deficient conditions in vivo using the hydrodynamic tail-vein injection system. An immunofluorescence analysis showed that c-MYC was localized in the nuclei, the FLAG-tagged Cldn7 (Cldn7^FLAG^) was localized in cytoplasm, and nucleoli in the hydrodynamically transfected hepatocytes at 2 dpi of pT3-c-MYC, pT3-Cldn7^FLAG^ and pX330-p53 (hep-c-MYC/p53KO/Cldn7^FLAG^) (Fig. 4D), confirming the co-expression of these two transgenes in the same cells. The co-expression of Cldn7^FLAG^ significantly diminished the hep-c-MYC/p53KO cancer development, and the overall tumor volume in the hep-c-MYC/p53KO/Cldn7^FLAG^ mice was equivalent to that of hep-c-MYC mice at 11 dpi (Fig. 4E, F). These results suggest that c-MYC activation of p53 leads to upregulation of Cldn7 and repression of c-MYC-mediated oncogenesis and overexpression of Cldn7 counteracts the accelerated oncogenesis by c-MYC in the absence of p53 (hep-c-MYC/p53KO). Further, our studies imply that AR-V7 and, to a lesser extent AR-FL in males, could bypass the p53 regulation and directly repress the Cldn7 tumor suppressor, thereby exacerbating the c-MYC-driven hepatic oncogenesis.

## Discussion

The high cancer heterogeneity of HCC is a major problem in developing effective therapeutic strategies for this deadly cancer [2]. Hence, it is crucial to identify the heterogeneously expressed factors that contribute to HCC initiation and progression. Our preliminary study demonstrated that AR-V7 was co-expressed with c-MYC in 38% clinical HCC cases (Supplementary Fig. 1C). Further, the in vivo analyses using the hydrodynamic tail-vein injection technique showed that AR-V7 exacerbated the c-MYC-mediated liver oncogenesis regardless of the sexes (Fig. 1). These results strongly suggest that AR-V7 is an important oncogenic promoter contributing to the HCC heterogeneity in male and female patients in a ligand-independent manner(s).

The full-length AR harboring the C-terminal ligand-binding domain (LBD) functions as a transcription regulator in a ligand-dependent manner [17, 43]. Since the prevalence of HCC is 3–5 folds higher in males than females [44], as the male sex hormone, androgen, and its full-length receptor AR-FL have been considered to constitute an important signaling pathway in mediating such sex differences. Indeed, various studies demonstrated that androgen/AR-FL exacerbated cell proliferation and liver cancer development under experimental conditions [45, 46]. However, clinical trials of antiandrogen therapy for unresectable HCC patients did not yield any significantly improved outcomes [47, 48]. Although cancer stage-dependent roles of AR have been suggested, the reasons for such inconsistency have not been well explained [49]. Noteworthy, a similar situation is well-known in prostate cancer. While most patients with localized and advanced prostate cancer initially respond to androgen deprivation therapy (ADT), many of them eventually evolve into metastatic castration-resistant prostate cancer that aggressively progresses into lethal cancer irrespective of the serum androgen level [16, 17, 50]. Recent studies revealed that the aberrantly spliced AR isoforms with truncation of the LBD, such as AR-V7, could partially contribute to such metastatic castration-resistant prostate cancer development [18, 19]. Although the correlation between the AR-V7 expression and HCC prognosis remains unclear, our results suggest a potential contribution of AR-V7 to HCC initiation and progression in a subpopulation(s) involving c-MYC oncogenesis. It would be important to evaluate the correlation between the AR-V7 expression and clinical features of HCC in future studies.

Either AR-V7 expression or AR-FL overexpression alone in hepatocytes did not result in tumorigenesis in the mouse liver (Supplementary Fig. 4), suggesting that these molecules are not capable to initiate hepatocarcinogenesis by themselves. As a grand orchestrator of oncogenic processes of a variety of cancers, the c-MYC gene is frequently activated and/or amplified in HCC. However, c-MYC overexpression alone in non- and pre-malignant cells frequently increases sensitivity to apoptosis [37, 38]. Interestingly, some in vivo studies demonstrated that hepatocytic overexpression of c-MYC alone in adult mice was not sufficient to initiate cancer or promote cancer after a prolonged latency [3, 10, 11]. The present study showed that AR-V7 could exacerbate in both sexes and AR-FL could exacerbate in a male-biased manner the c-MYC oncogenic actions in HCC development (Fig. 1), suggesting that AR-V7 and AR-FL promote the c-MYC-driven oncogenesis, thereby contributing to the heterogeneity and sexual dimorphisms in HCC initiation and progression respectively. Noteworthy, the impact of AR-V7 on the c-MYC-driven HCC in female mice was more significant than those of AR-FL in male mice (Fig. 1), indicating the importance of AR-V7 in the heterogeneity of HCC regardless of sexes. Further, since aberrant c-MYC activation/mutations have been associated with many cancer types [4, 5], hence in addition to prostate and liver cancers, the detection of AR-V7 in other human cancers, such as breast, bladder, and ovarian cancers (Supplementary Fig. 1B), suggests the existence of potential AR-V7 exacerbation on c-MYC oncogenesis in these AR-V7-positive tumors.

**Fig. 5:**
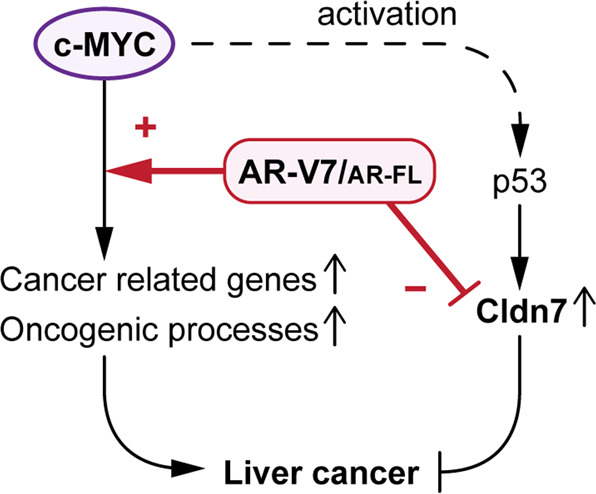
A schematic diagram illustrating the likely mechanisms of AR-V7 and to a lesser extent AR-FL exacerbate the c-MYC-driven HCC. AR-V7 potentiates the cancer-related genes and oncogenic processes regulated by c-MYC, while it represses a tumor suppressor Cldn7 that is upregulated by c-MYC-overexpression via its activation of the p53 signaling pathway. AR-FL partly exerts similar functions to those of AR-V7 in the presence of androgen in males.

The transcriptome and bioinformatics analyses revealed that the AR-V7 co-expression further altered the c-MYC-cancer cell-intrinsic biological processes, including proliferation, protein, and ribosomal biosynthesis, gene instability, and metabolism in hepatocarcinogenesis (Fig. 2B, C). Importantly, AR-V7 could alter gene expression of the c-MYC-regulated lipid metabolism and steroid/sterol metabolism (Fig. 2B, C). Altered lipid metabolism has been strongly associated with the onset and progression of HCC [51–53]. In particular, fatty acid synthase has been demonstrated to play important role in c-MYC-driven hepatocarcinogenesis [53, 54]. Our results suggest that AR-V7, and partially AR-FL, could potentiate the oncogenic functions of c-MYC in these and other oncogenic processes (Fig. 5). Interestingly, we found that AR-V7 also suppressed a tumor suppressor Cldn7 that was upregulated by c-MYC overexpression via the p53 signaling pathway (Fig. 4). It has been reported that the hepatocytic c-MYC expression in adult mice could activate the p53 signaling pathway [11]. Importantly, other pathways, which inhibit the p53 signaling pathway, such as AKT and YAP/TAZ pathways, could synergistically exacerbate c-MYC oncogenesis [55, 56]. Hence, suppression of the p53 signaling pathway could be a crucial and common mechanism(s) in promoting c-MYC-mediated hepatocarcinogenesis. Our findings suggest that AR-V7 and partially AR-FL could inhibit the downstream tumor suppressor(s), i.e. Cldn7, in the p53 signaling pathway, thereby exacerbating the c-MYC-driven hepatocarcinogenesis.

Our study demonstrated that AR-V7 and AR-FL (at a lesser level and male-specific manner) exacerbate the c-MYC-mediated hepatocarcinogenesis by potentiating the oncogenic and minimizing the anti-proliferative functions of c-MYC in the liver (Fig. 5), thereby providing critical insights into the molecular mechanisms partially responsible for the heterogeneity in HCC. Future studies to elucidate the molecular mechanisms of how AR-V7 could regulate the metabolic processes and p53 signaling pathway will provide important clues in identifying treatment targets for this deadly cancer. Importantly, the current development of AR-V7-specific drugs in treatments of metastatic castration prostate cancer could offer potential resources for translational applications in therapeutic strategies on the AR-V7-positive subpopulation of HCC patients.

## Supplementary information

Supplementary Figures

Supplementary Table 1

Supplementary Table 2

Supplementary Table 3

Supplementary Table 4

## Data Availability

The RNA-seq data will be deposited in the GEO Database with accession numbers upon acceptance and publication of this manuscript.
