# Supplementary material for: Androgen receptor variant 7 exacerbates hepatocarcinogenesis in a c-MYC-driven mouse HCC model
Source: Oncogenesis. 2023 Feb 6;12(1):4. doi: 10.1038/s41389-023-00449-3 (PMC9902460; doi:10.1038/s41389-023-00449-3)
Supplement: Supplementary file 1 — Supplementary Figures [file 41389_2023_449_MOESM1_ESM.docx]

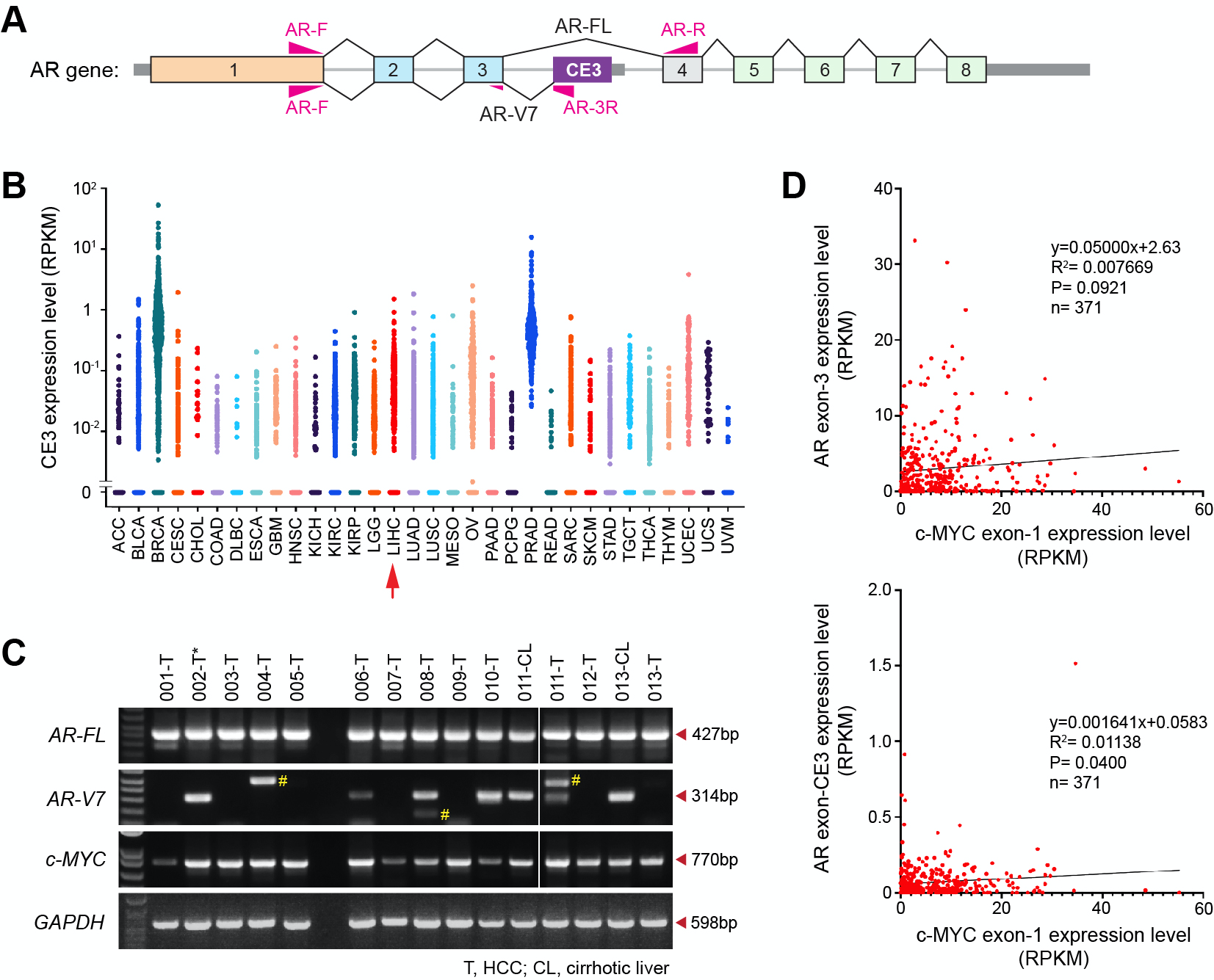


**Supplementary Fig. 1 AR-V7 is heterogeneously expressed in HCC cases.** **A** Schematic representation of the structure of the human androgen receptor (AR) gene. The splicing patterns for the full-length AR (AR-FL) and AR-V7 variant, are illustrated as marked. Arrows indicate the positions of primers used in the RT-PCR analyses (results shown in C) for these AR transcripts. **B** Expression levels of the AR-V7-specific exon CE3 in pan-cancer data of TCGA datasets. Arrow indicates liver hepatocellular carcinoma (LIHC). BRCA, breast invasive carcinoma; PRAD, prostate adenocarcinoma. A full list of abbreviations and sample sizes are summarized in Supplementary Table 1. **C** Results of conventional RT-PCR analyses for the expressions of AR-FL, AR-V7, c-MYC, and GAPDH in clinical HCC samples (T) and cirrhotic liver samples (CL). #, non-specific PCR products. AR-V7 was detectable in selected samples confirming the previous TCGA datamining findings [23]. All AR-V7 specific bands were confirmed by DNA sequencing. Primer sequences for respective genes are shown in Supplementary Table 3. **D** Scatter plot of c-MYC and AR exon-3 expression levels (top), and c-MYC and AR exon-CE3 expression levels (bottom) from 371 HCC patient samples in TCGA cohort. Values indicate reads per kilobase million (RPKM) by RNA-seq analyses. The equation of regression line, R^2^ value, and P value for non-zero slope in linear regression analysis using Prism 9 software are indicated. While there was no correlation between the expression levels of c-MYC and AR exon-3 (top), there was a weak correlation between the expression levels of c-MYC and AR exon-CE3 (bottom).

**
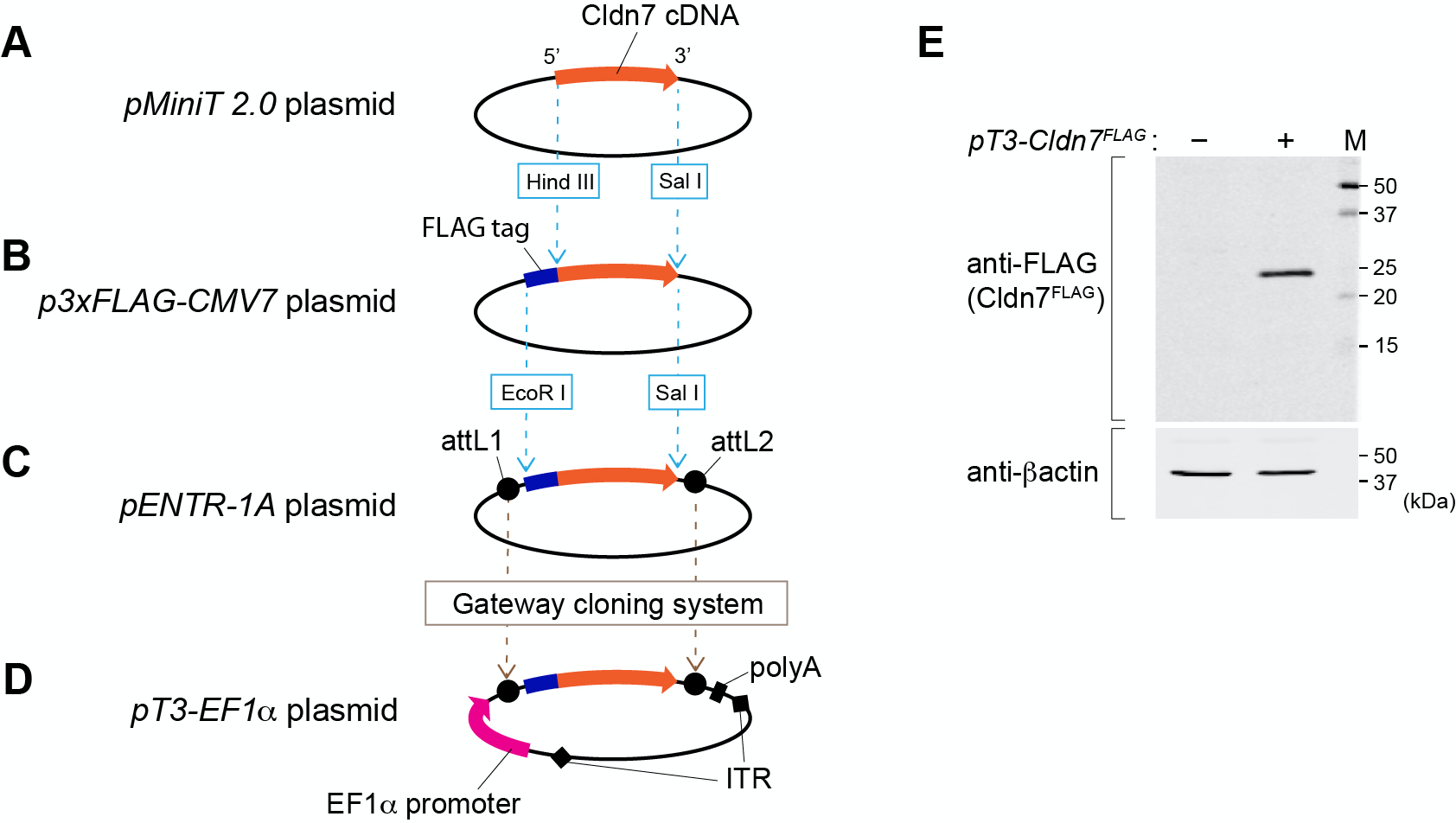
**

**Supplementary Fig. 2 A−D** Workflow of construction of pT3-Cldn7^FLAG^. The mouse Cldn7 cDNA was generated by RT-PCR, cloned into a cloning vector pMiniT 2.0 (New England Biolabs, Ipswich, MA), and confirmed by DNA sequencing (A). The DNA fragment encoding Cldn7 was excised using *Hind*III and *Sal*I and inserted between the *Hind*III and *Sal*I sites of p3xFLAG-CMV7 (Sigma-Aldrich) to add the FLAG-tag coding sequences at the 5’-end (B). The DNA fragment coding for the FLAG-tagged Cldn7 (Cldn7^FLAG^) was excised using *Eco*RI and *Sal*I, and inserted between the *Eco*RI and *Sal*I sites of pENTR-1A (Invitrogen, Thermo Fisher Scientific, Waltham, MA). The DNA fragment encoding Cldn7^FLAG^ was then inserted into the cloning site of the mammalian cell expression vector pT3-EF1α using the Gateway cloning system (Thermo Fisher Scientific) (D). attL1 and attL2, recombination sites for the Gateway LR clonase; ITR, inverted terminal repeat; polyA, polyadenylation signals. **E** Expression of the Cldn7^FLAG^ in the 293T cells transiently transfected with pT3-Cldn7^FLAG^ was verified by a Western blot using anti-FLAG rabbit polyclonal antibody (Sigma-Aldrich #F7425) and anti-βactin mouse monoclonal antibody (clone AC-15, Sigma-Aldrich #A1978). M, molecular size marker; -, control cells without transfection; +, cells transfected with pT3-Cldn7^FLAG^. Transfection and Western blot were performed as described previously [*Kido et al., 2011, PLoS ONE, 6(7), e22979*].

**
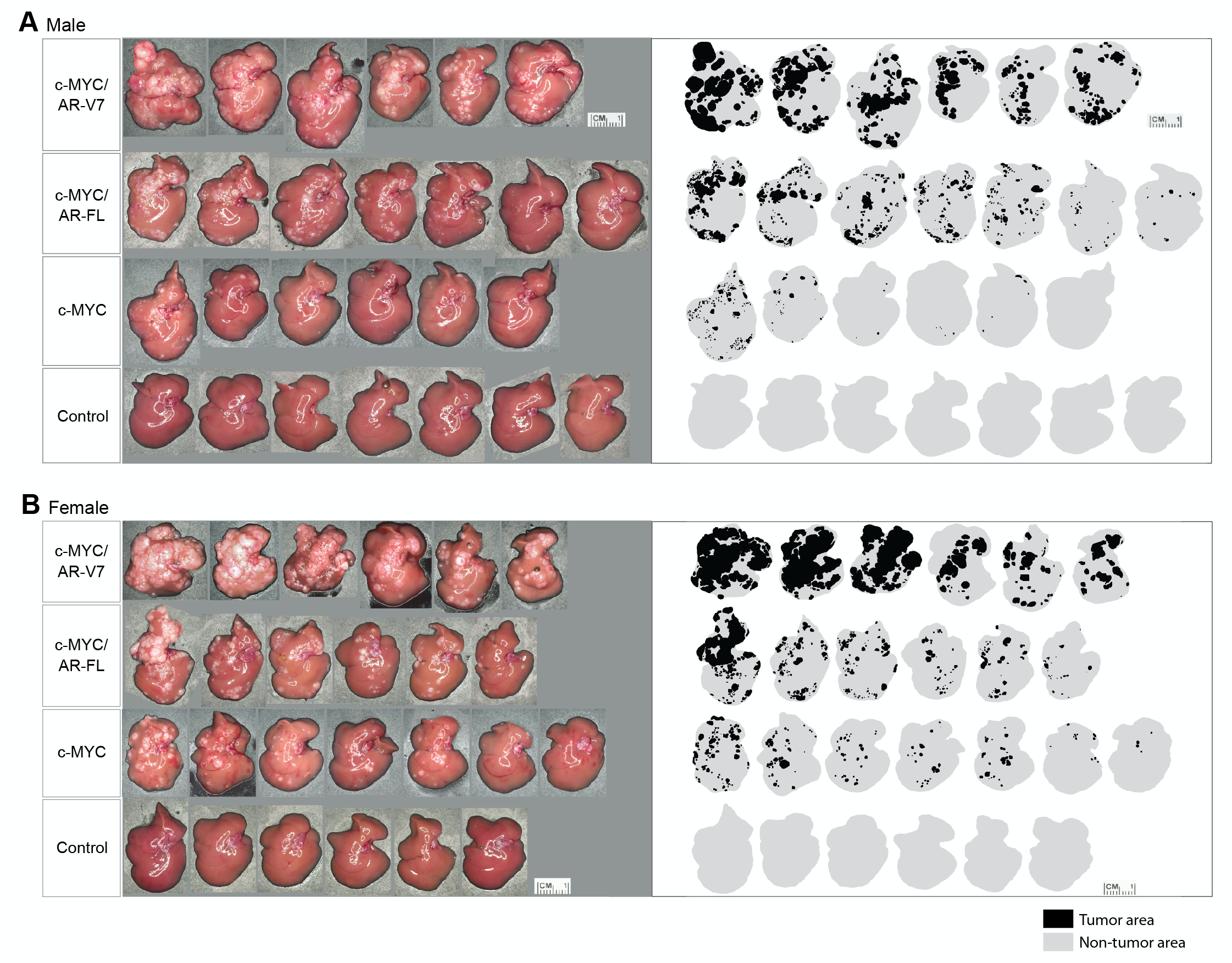

Supplementary Fig. 3** (Left panels) Macroscopic images of the liver in male mice (A) and female mice (B) at 20 dpi of the indicated groups. (Right panels) Corresponding images of the tumor areas (black) and non-tumor areas (gray) were manually selected for measurements by the NIH ImageJ program, see Materials and Methods.

**
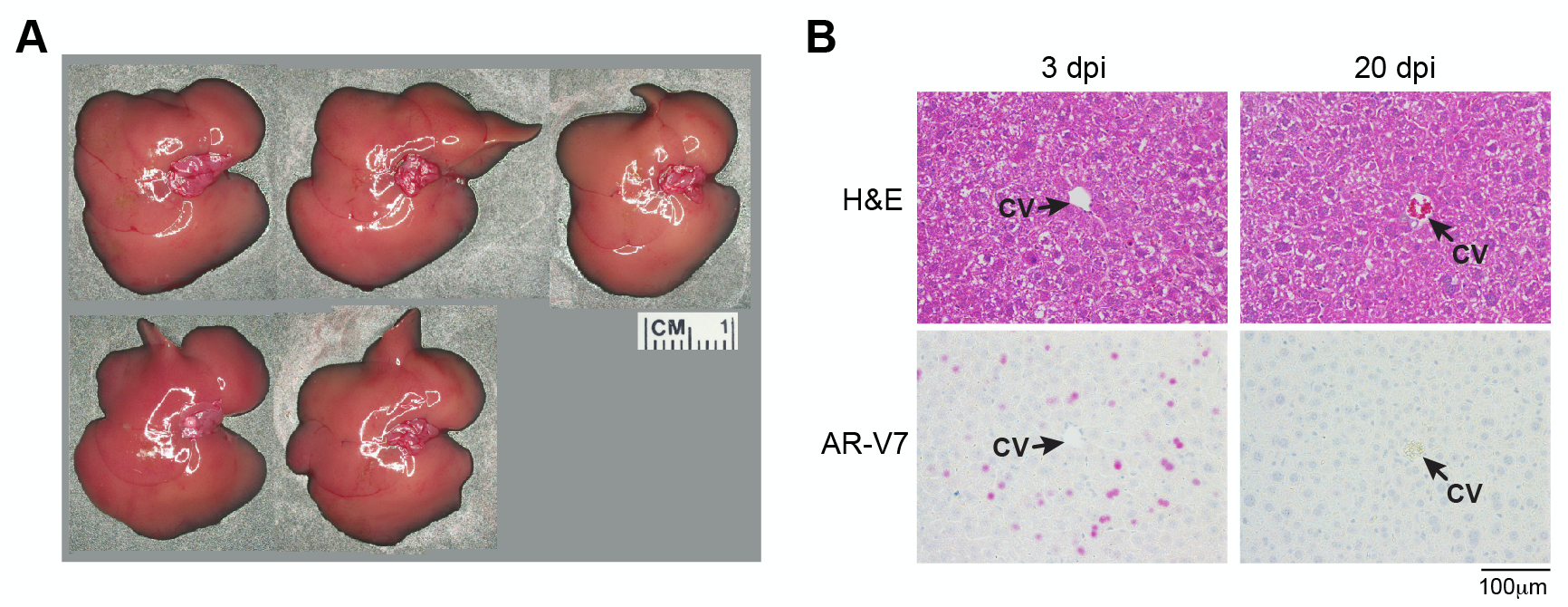
**

**Supplementary Fig. 4** Macroscopic phenotype of the livers in 5 male mice at 20 days post-injection (dpi) of pT3-AR-V7 with pCMV-SB100. No tumor development was observed in any of these mice. **B** Microscopic images of H&E-stained tissue section (top) and immunohistochemistry-stained sections with anti-AR-V7 antibody (bottom) for liver samples at 3 and 20 dpi of pT3-AR-V7 with pCMV-SB100, showing AR-V7 expression (red) was detected in liver samples of 3 dpi but not 20 dpi animals. Nuclei were counter-stained by hematoxylin (blue). CV, central vein.

**
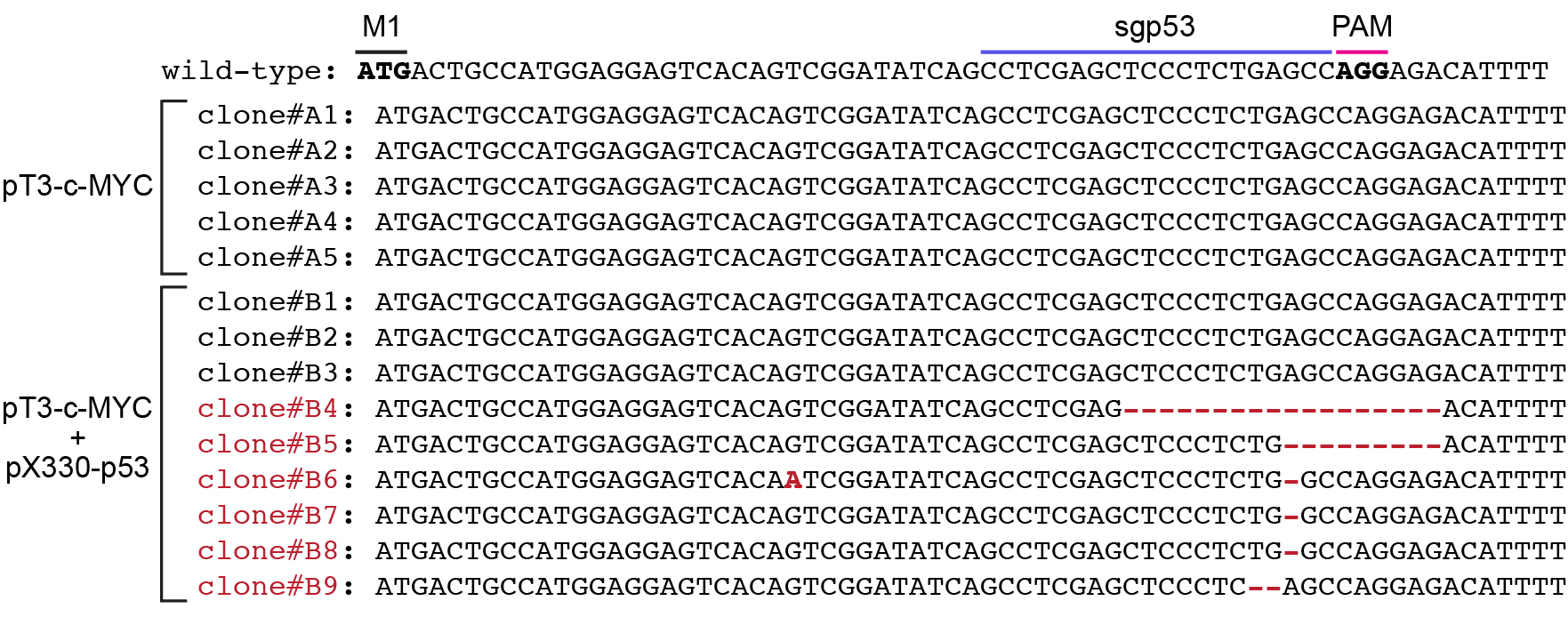
**

**Supplementary Fig. 5** Results of sequencing analysis of the p53 transcripts expressed in the liver cancer induced by hydrodynamic injection of pT3-c-MYC alone (5 clones) or pT3-c-MYC and pX330-p53 (9 clones). M1, the translation initiation codon; PAM, protospacer adjacent motif sequences; sgp53, sequences of guide RNA; -, deletion. No mutations were found in the p53 transcripts expressed in tumors promoted by pT3-c-MYC alone (clones #A1-A5). In contrast, 6 out of 9 clones from tumors promoted by pT3-c-MYC and pX330-p53 harbored in-frame or frame-shift mutations within the region covered by the guide RNA (clones #B4-B9). Results indicated that pX330-p53 was successful in inactivating the endogenous p53 gene in the c-MYC/p53KO mice.
