# Supplementary material for: Androgen receptor variant 7 exacerbates hepatocarcinogenesis in a c-MYC-driven mouse HCC model
Source: Oncogenesis. 2023 Feb 6;12(1):4. doi: 10.1038/s41389-023-00449-3 (PMC9902460; doi:10.1038/s41389-023-00449-3)
Supplement: Supplementary file 2 — Supplementary Table 1 [file 41389_2023_449_MOESM2_ESM.docx]

**Supplementary Table 1** Abbreviations and sample sizes of TCGA pan-cancer gene expression datasets.

| Abbreviation | Cancer type | Sample size |
| --- | --- | --- |
| ACC | Adrenocortical carcinoma | 79 |
| BLCA | Bladder Urothelial Carcinoma | 407 |
| BRCA | Breast invasive carcinoma | 1095 |
| CESC | Cervical squamous cell carcinoma and endocervical adenocarcinoma | 302 |
| CHOL | Cholangiocarcinoma | 36 |
| COAD | Colon adenocarcinoma | 283 |
| DLBC | Lymphoid Neoplasm Diffuse Large B-cell Lymphoma | 48 |
| ESCA | Esophageal carcinoma | 184 |
| GBM | Glioblastoma multiforme | 153 |
| HNSC | Head and Neck squamous cell carcinoma | 520 |
| KICH | Kidney Chromophobe | 66 |
| KIRC | Kidney renal clear cell carcinoma | 533 |
| KIRP | Kidney renal papillary cell carcinoma | 290 |
| LGG | Brain Lower Grade Glioma | 515 |
| LIHC | Liver hepatocellular carcinoma | 371 |
| LUAD | Lung adenocarcinoma | 515 |
| LUSC | Lung squamous cell carcinoma | 501 |
| MESO | Mesothelioma | 87 |
| OV | Ovarian serous cystadenocarcinoma | 304 |
| PAAD | Pancreatic adenocarcinoma | 178 |
| PCPG | Pheochromocytoma and Paraganglioma | 179 |
| PRAD | Prostate adenocarcinoma | 497 |
| READ | Rectum adenocarcinoma | 93 |
| SARC | Sarcoma | 259 |
| SKCM | Skin Cutaneous Melanoma | 104 |
| STAD | Stomach adenocarcinoma | 415 |
| TGCT | Testicular Germ Cell Tumors | 134 |
| THCA | Thyroid carcinoma | 505 |
| THYM | Thymoma | 120 |
| UCEC | Uterine Corpus Endometrial Carcinoma | 176 |
| UCS | Uterine Carcinosarcoma | 57 |
| UVM | Uveal Melanoma | 80 |
