# Supplementary material for: Androgen receptor variant 7 exacerbates hepatocarcinogenesis in a c-MYC-driven mouse HCC model
Source: Oncogenesis. 2023 Feb 6;12(1):4. doi: 10.1038/s41389-023-00449-3 (PMC9902460; doi:10.1038/s41389-023-00449-3)
Supplement: Supplementary file 3 — Supplementary Table 2 [file 41389_2023_449_MOESM3_ESM.docx]

**Supplementary Table 2** TaqMan mouse gene expression assay kit IDs and amplicon size. The primer sequences are proprietary by Thermo Fisher Scientific.

| Organism | Gene | Assay ID Amplicon size (bp) |
| --- | --- | --- |
| Mouse | *B4galnt1* | Mm01135934_g1 139 |
|  | *Cldn7* | Mm00516817_m1 86 |
|  | *Ffar4* | Mm00725193_m1 79 |
|  | *Gapdh* | Mm99999915_g1 109 |
|  | *Il1rl2* | Mm00519245_m1 73 |
|  | *Trp53* | Mm01731290_g1 119 |
