# Supplementary material for: Androgen receptor variant 7 exacerbates hepatocarcinogenesis in a c-MYC-driven mouse HCC model
Source: Oncogenesis. 2023 Feb 6;12(1):4. doi: 10.1038/s41389-023-00449-3 (PMC9902460; doi:10.1038/s41389-023-00449-3)
Supplement: Supplementary file 4 — Supplementary Table 3 [file 41389_2023_449_MOESM4_ESM.docx]

**Supplementary Table 3** Primer sequences used for the RT-PCR analysis.

| Organism | Gene | Sequences | Binding site | Amplicon size (bp) |
| --- | --- | --- | --- | --- |
| Human | *AR-FL* | 5’-CTACTCCGGACCTTACGGGGACA-3’ | Exon-1 | 427 |
|  |  | 5’-GGGCTGACATTCATAGCCTTCAA-3’ | Exon-4 |  |
|  | *AR-V7* | 5’-CTACTCCGGACCTTACGGGGACA-3’ | Exon-1 | 314 |
|  |  | 5’-TGCCAACCCGGAATTTTTCTCCC-3’ | Exon-3 (5 nt) and exon-CE3 (18 nt) |  |
|  | *GAPDH* | 5’-CCACCCATGGCAAATTCCATGGCA-3’ | Exon-4 | 598 |
|  |  | 5’-TCTAGACGGCAGGTCAGGTCCACC-3’ | Exon-8 |  |
|  | *c-MYC* | 5’-ACCTCCAGCTTGTACCTGCAGGATCTG-3’ | Exon-2 | 804 |
|  |  | ﻿5’-TTACGCACAAGAGTTCCGTAGCTGTTC-3’ | Exon-3 |  |
| Mouse | *Trp53* | 5’-ATTCAGGCCCTCATCCTCCT-3’ | Exon-1 | 194 |
|  | (*p53*) | 5’-AGCAACAGATCGTCCATGCA-3’ | Exon-1 |  |
